# Importance of Veins for Neurosurgery as Landmarks Against Brain Shifting Phenomenon: An Anatomical and 3D-MPRAGE MR Reconstruction of Superficial Cortical Veins

**DOI:** 10.3389/fnana.2020.596167

**Published:** 2020-12-15

**Authors:** Santino Ottavio Tomasi, Giuseppe Emmanuele Umana, Gianluca Scalia, Roberto Luis Rubio-Rodriguez, Pier Francesco Cappai, Crescenzo Capone, Giuseppe Raudino, Bipin Chaurasia, Maurizio Salvati, Nicolas Jorden, Peter A. Winkler

**Affiliations:** ^1^Department of Neurological Surgery, Christian Doppler Klinik, Salzburg, Austria; ^2^Paracelsus Medical University, Salzburg, Austria; ^3^Laboratory for Microsurgical Neuroanatomy, Christian Doppler Klinik, Salzburg, Austria; ^4^Department of Neurosurgery, Trauma Center, Gamma Knife Center, Cannizzaro Hospital, Catania, Italy; ^5^Neurosurgery Unit, Highly Specialized Hospital and of National Importance “Garibald”, Catania, Italy; ^6^Skull Base and Cerebrovascular Laboratory, University of California, San Francisco, San Francisco, CA, United States; ^7^Department of Neurological Surgery, University of California, San Francisco, San Francisco, CA, United States; ^8^Department of Otolaryngology- Head and Neck Surgery, University of California, San Francisco, San Francisco, CA, United States; ^9^Department of Neurosurgery, Azienda Ospedaliera G. Brotzu, Universitá degli Studi di Sassari, Sassari, Italy; ^10^Department of Peripheral Nerve Surgery, Azienda Unità Sanitaria Locale Romagna, Ospedale Civile di Faenza, Faenza, Italy; ^11^Department of Neurosurgery, Istituto di Ricovero e Cura ad Alta Specializzazione Policlinico di Monza, Monza, Italy; ^12^Department of Neurosurgery, Neurosurgery Clinic, Birgunj, Nepal; ^13^Department of Neurosurgery, Policlinico Tor Vergata, Rome, Italy; ^14^Radiologie und Nuklearmedizin Dachau, Karlsfeld, Germany

**Keywords:** superficial cortical veins, brain shift, 3D reconstruction, MPRAGE MR-sequences, microsurgical neuroanatomy

## Abstract

Modern neurosurgery uses preoperative imaging daily. Three-dimensional reconstruction of the cortical anatomy and of the superficial veins helps the surgeons plan and perform neurosurgical procedures much more safely. The target is always to give the patient maximum benefit in terms of outcome and minimize intraoperative and postoperative complications. This study aims to develop a method for the combined representation of the cerebral cortex anatomy and the superficial cerebral veins, whose integration is beneficial in daily practice. Only those patients who underwent surgical procedures with craniotomy and a large opening of the dura mater were included in this study, for a total of 23 patients, 13 females (56.5%) and 10 males (43.5%). The average age was 50.1 years. We used a magnetic resonance tomograph Magnetom Vision® 1.5T (Siemens AG). Two sequences were applied: a strongly T1-weighted magnetization-prepared rapid acquisition with gradient echo (MPRAGE) sequence to visualize cerebral anatomical structures, and a FLASH-2D-TOF angiography sequence to visualize the venous vessels on the cortical surface after the administration of a paramagnetic contrast agent. The two data sets were superimposed manually, co-registered in an interactive process, and merged to create a combined data set, segmented and visualized as a three-dimensional reconstruction. Furthermore, we present our method for visualizing superficial veins, which helps manage brain shift (BS). We also performed anatomical observations on the reconstructions. The reconstructions of the cortical and venous anatomy proved to be a valuable tool in surgical planning and positively influenced the surgical procedure. Due to the good correlation with the existing surgical site, this method should be validated on a larger cohort or in a multicentric study.

## Introduction

Modern neurosurgery uses preoperative imaging daily. Three-dimensional reconstruction of the cortical anatomy and of the superficial veins helps the surgeons plan and perform neurosurgical procedures and minimize complications. This study aims to develop a method for the combined representation of the cerebral cortex anatomy and the superficial cerebral veins, whose integration is beneficial in daily practice. Two sequences were applied: a strongly T1-weighted magnetization-prepared rapid acquisition with gradient echo (MPRAGE) sequence to visualize cerebral anatomical structures, and a FLASH-2D-TOF angiography sequence to visualize the venous vessels on the cortical surface after the administration of a paramagnetic contrast agent. The two data sets were superimposed manually, co-registered in an interactive process, and merged to create a combined data set, then segmented and visualized as a three-dimensional reconstruction. We provide photo documentation of eachpatient's surgical site. We present our method for visualizing superficial veins, which helps manage brain shift (BS), as the veins on the cortical surface shift together with the underlying brain tissue and therefore do not—or only minimally—change their relative location to the cortical landmarks. Our study includes x-rays, computer tomography, magnetic resonance tomography, and nuclear medicine examinations, as well as ultrasound images and photographs of the surgical site in the case of a previous surgical intervention. We also performed anatomical observations on the reconstructions. The reconstructions of the cortical and venous anatomy proved to be a valuable tool in surgical planning, and it influenced the surgical procedure positively.

## Materials and Methods

### Patient Population

**Figure 1:**
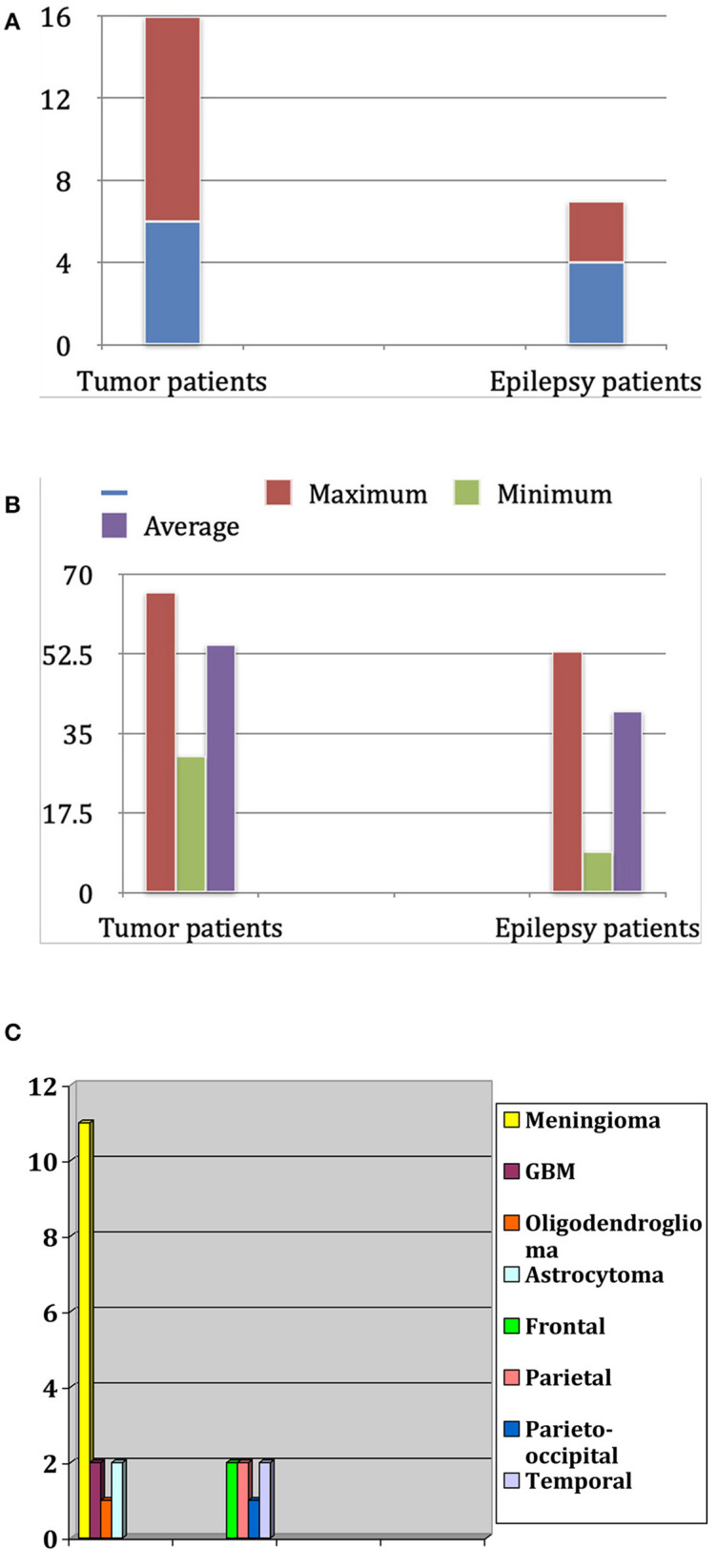
**(A)** Distribution of patients for sex and diagnosis. **(B)** Age distribution in years in both groups (tumor patients and epilepsy patients). **(C)** Frequency of tumor types in the tumor patients group and localization of epileptogenic focus in the epilepsy patients group.

Our study is an anatomic, observational study, conducted exclusively in the clinic and laboratory of the first (S.O.T.) and senior (P.A.W.) authors, who also performed the anatomical and surgical procedures. The study includes only those patients who underwent surgical procedures with craniotomy and a large opening of the dura mater. Twenty-three patients were enrolled, 13 females (56.5%) and 10 males (43.5%). The average age was 50.1 years. We divided the patient population into two groups (Figure 1A). The first group consisted of 16 patients affected by brain tumors, 11 females and five males. The average age in this first group was 54.5 years, with a minimum age of 30 and a maximum of 66. The second group, composed of three female and four male patients, had pharmacoresistant focal epilepsy. Their age ranged between 9 and 53 years, with a mean of 40.0 years (Figure 1B). Knowledge of the location of important blood vessels—especially veins—is of great significance, both in planning the surgery itself and implanting subdural electrodes, as cortical stimulation entails the risk of venous complications.

### Brain Shift

We compared the preoperative reconstruction of the superficial cortical vessels with intraoperative photographs of the cortical surface. For the comparative evaluation of the two representations, it is crucial to consider brain shift, which is the deformation of brain tissue during, and due to, neurosurgical intervention (Holmes and Sargent, 1915; Merwarth, 1942; Huber, 1982; Seeger, 1984; Rhoton, 1989, 2002; Koperna et al., 1992; Apuzzo, 1993; Sakaki et al., 1995; Roberts et al., 1998; Choi et al., 2004). Besides obvious causes, such as the resection of tissues or the drainage of CSF from the ventricles, cerebral blood volume (CBV)—depending on carbon dioxide (CO_2_) concentration and/or administration of osmotic agents (e.g., Mannitol), anesthetics or steroids—also plays an important role in the brain shift process. In some cases, an initial swelling of the brain tissue occurs shortly after the opening of the dura mater, without signs of increased intracranial pressure.

Moreover, after removing a brain tumor, there is always a distortion of the brain tissue. Therefore, a brain shift of some centimeters can occur. This situation usually shows a discrepancy between the preoperative navigation imagines and the “real-time” situation in the operating room. Multiple intraoperative MRIs can control this dynamic process. Patient population and related pathologies are summarized in Figure 1C.

### Magnetic Resonance Imaging

We used a magnetic resonance tomograph Magnetom Vision® 1.5T (Siemens AG) (Andeweg, 1996; Siemens, 2000). We examined the patients with two different sequences: MPRAGE sequences, which are TurboFLASH sequences extended to 3D and allow high-contrast imaging of the brain, were acquired natively, and FLASH-2D-TOF angiography, to visualize the venous vessels in as high a contrast as possible (Perese, 1958; Stephens and Stilwell, 1969; Strother et al., 1994; Stevenson et al., 1995; Imai et al., 1996; Hunerbein et al., 1997; Liang et al., 2001; Immonen et al., 2004). We oriented the layers to be as perpendicular to the brain surface as possible in the relevant area.

### Overlay, Co-registration, and Processing of Records

The MRI data were transferred to a VoxelQ workstation (Picker International, Cleveland, OH, USA) for further processing. The MPRAGE sequence was defined as the primary data set and the FLASH-2D-TOF angiography as the secondary data set (active data set).

### Interactive Overlay of the Data Sets

**Figure 2:**
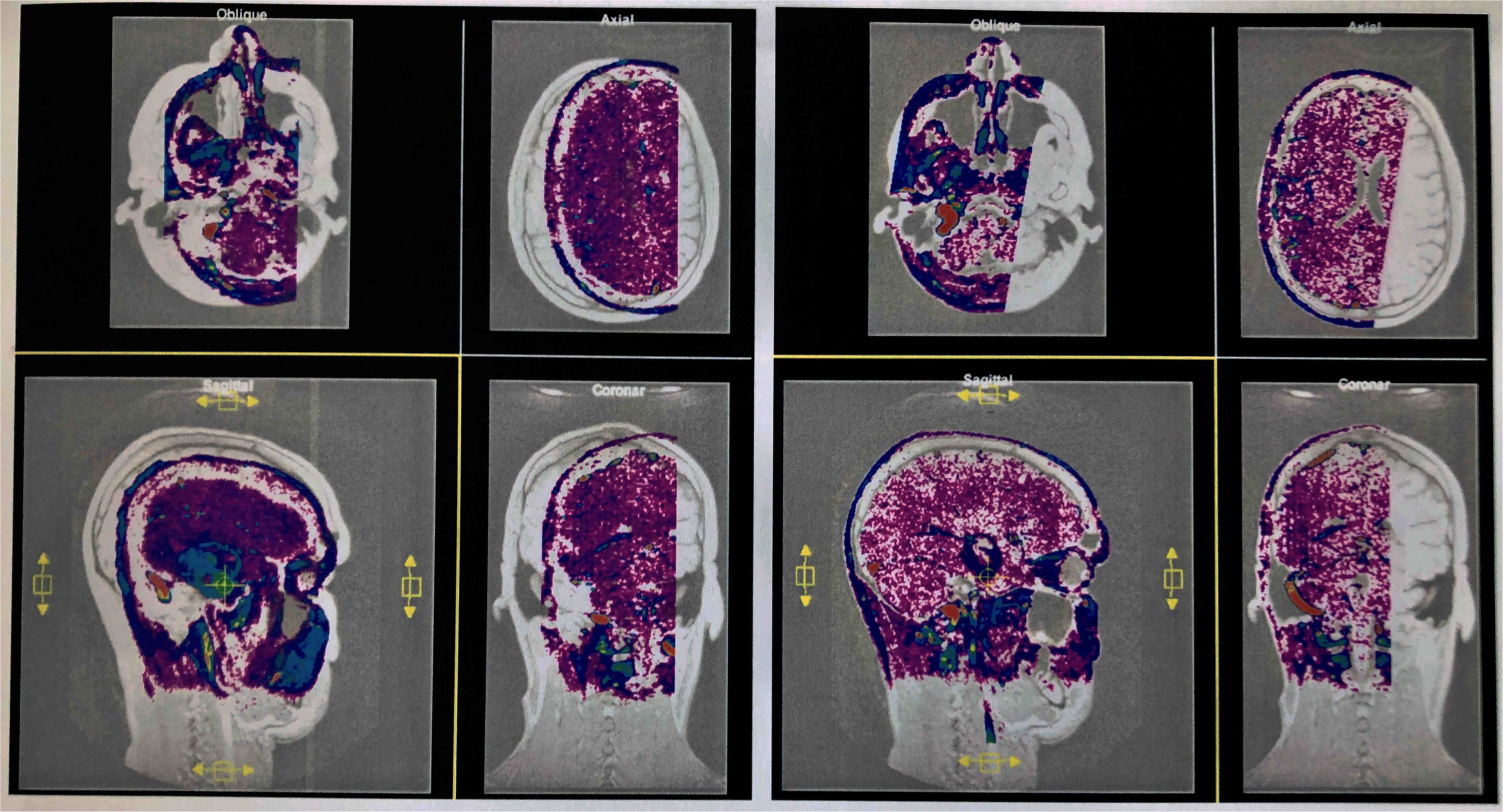
Interactive overlay of the data sets. Representation of the primary and secondary data sets in the interactive module of VoxelQ. The active dataset can be moved and rotated in the selected window (yellow border). The views are updated automatically and immediately in all windows (**left**). The secondary data set has been aligned with the primary data set by displacements in different planes and rotations about different axes (successful co-registration) (**right**).

The active data set was color-coded and transparently superimposed onto the primary data set. We assigned a color to each gray value of the active data set. We were interested in larger venous and other non-vascular—structures, especially the SSS, transverse sinus, rectus sinus, larger cortical and bridging veins, the vessels of the interhemispheric gap, and the tentorium cerebelli (Figure 2).

### Co-registration of the Data Sets and Adjustment of the Threshold Value

In the *Combiner* module of the VoxelQ, we merged the two data sets into a single, combined data set. Before we created the new data set, however, we increased the contrast of the secondary data set to a maximum so that the pixels above a specific brightness value would be displayed in monochrome blue, while the pixels with a brightness value below this threshold would not be displayed at all.

### Segmentation of the Data Set

**Figure 3:**
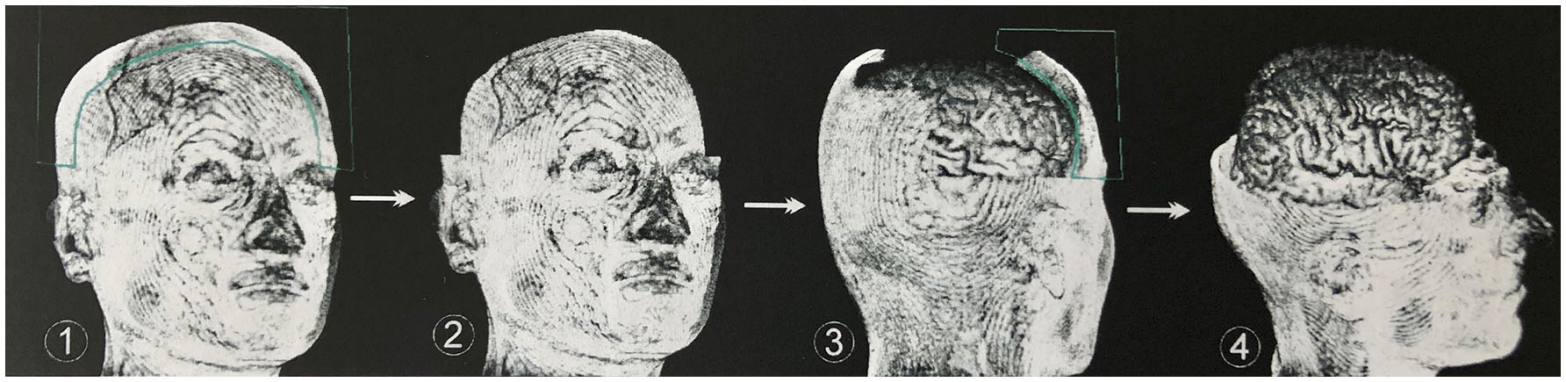
Procedure for volume sculpting. Marked extracerebral structures (1) are cut away perpendicularly to the viewing plane (2). Rotation of the data set around one or more axes again results in well-exposed extracerebral structures, which are marked (3) and removed. This process is repeated until undisturbed supervision on the cerebrum is possible (4). In most cases, this was done using the *Volume Sculpting* module of the VoxelQ workstation (Amira, Indeed-Visual Concepts GmbH, Berlin, Germany).

To obtain an unobstructed view of the cortical surface and the vessels running on it, the overlaying structures—i.e., cranium, and, if possible, dura mater—must be removed from the data volume. In most cases, this was done using the *Volume Sculpting* module of the VoxelQ workstation. We used a commercial PC with Amira (Indeed—Visual Concepts GmbH, Berlin, Germany). The combined data set is displayed on the screen; the parts to be removed are marked and deleted from the data volume. Since the parts of the 3D data set to be deleted are marked on a two-dimensional projection, the data set must be rotated successively about one or more axes in a process similar to peeling an apple (Figure 3).

**Figure 4:**
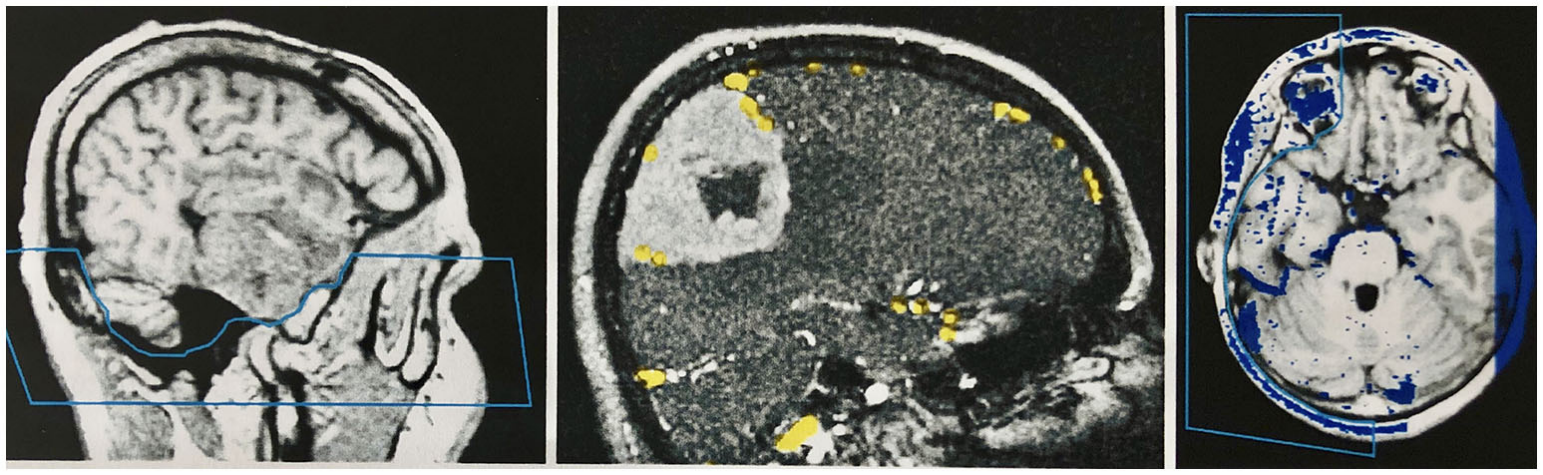
Manual segmentation in the two-dimensional slice images of the data sets. **Left**: Segmentation of the skull base in the primary data set (MPRAGE sequence) by marking and removing the unwanted region before combining the records. **Middle**: Segmentation of the secondary data set (venous MR angiography) in a patient with a convexity meningioma. The veins are marked with a stamping tool. Only the marked voxels are later combined with the primary data set. A safe demarcation of the veins from the contrast medium-absorbing tumor is therefore possible. **Right**: Segmentation of the combined data set in the temporal area, to create an undisturbed view of the cortical surface in the 3D reconstruction of this region.

The veins are difficult to separate from the adjacent structures that have been removed, especially in the parasagittal and temporal regions. In particularly difficult cases, we used a different segmentation method, with which we divided the undesired structures from those desired in the 2D layer images before creating the 3D reconstruction (Figure 4). This manual segmentation method—which, however, requires more time—was particularly useful in those cases where veins ran near contrast-absorbing tumors, or where segmentation by volume sculpting did not lead to a satisfactory result. Depending on the goal, segmentation took place either in the primary data set, in the secondary data set, or in the combined data set. In our study, the tumor was often also segmented to hide it later in the combined data set or display it in a color-coded manner.

### Determination of the Geometric Error of the Interactive Co-registration

Since there is no binding gold standard, and external reference systems are themselves subject to errors, we examined the reproducibility of co-registration as a measure of the accuracy achieved. To determine the error in the interactive co-registration, we assumed that the secondary data set's position in relation to the primary data set would statistically fluctuate around the correct position after repeated geometric correlation. To obtain a gold standard for the secondary data set's correct position, we registered the morphological and angiographic MR data sets of three patients in 10 consecutive trials. We extracted the parameters of the absolute spatial position and orientation of the active data set from all the individual results. The geometric center of the 10 individual positions was determined, and it served as the gold standard against which we compared the results of the individual experiments.

To calculate the deviation from the individual overlay results' gold standard, we started by limiting the image volume to an average adult's cerebral cortex volume relevant to our study. We calculated this volume by applying a three-dimensional binary mask, which we obtained by increasing the contrast from an FDG-PET examination of a healthy adult's cortex. The image volume thus limited was then determined with the help of a Monte Carlo simulation, using 5,000 random pixels (Duvernoy et al., 1981). Then, we calculated the expected value of the normalized volume integral between the source and the image points of the geometric transformation applied by us, and the average value of the spatial distance between each of the 5,000 corresponding pixels of the gold standard and a single overlay experiment. Here we considered the specific properties of the VoxelQ workstation we used, such as the sequence of the rotations, axis orientation, and direction of rotation. The selection of 5,000 points proved to be sufficiently accurate (variation < 0.01 mm) to make representative statements for the entire image volume. The mean value of the spatial error results, obtained from the 10 registration experiments performed on one patient, indicates the average error—for this patient—of the interactive registration method used by us. To avoid influencing this result by selecting a patient with conditions favorable to superimposition (e.g., good quality MR imaging), we calculated this mean value for three randomly selected patients. The three values were averaged, and the result would correspond to the average spatial deviation of our procedure for one operator (intraobserver variability).

We also investigated the extent to which the registration method's spatial error depends on the user and their previous knowledge. For this purpose, we had five test operators each perform five overlays of the two data sets of a randomly selected patient. Two of the operators were well acquainted with the methodology of interactive overlapping used by us, one had little experience with it, and the other two had no previous training or experience in this field. The test subjects with little or no prior knowledge received a brief introduction to the software and the anatomical landmarks useful for the superposition, but they received no assistance in the actual geometric alignment of the data sets during the co-registration process. With the same method described above, we determined the average spatial error for each subject. The mean value over all five test subjects would correspond to our method's expected geometric error when performed by any one operator, i.e., experts and laypersons alike (interobserver variability).

### Time Required for Interactive Co-registration

To allow for a comparison with other data sets co-registration methods, we measured the time required for the method we used. The timing began at the opening of the VoxelQ workstation's interactive module, after both data sets had been loaded into the workspace, and ended when the geometric correlation was completed to our satisfaction.

**Figure 5:**
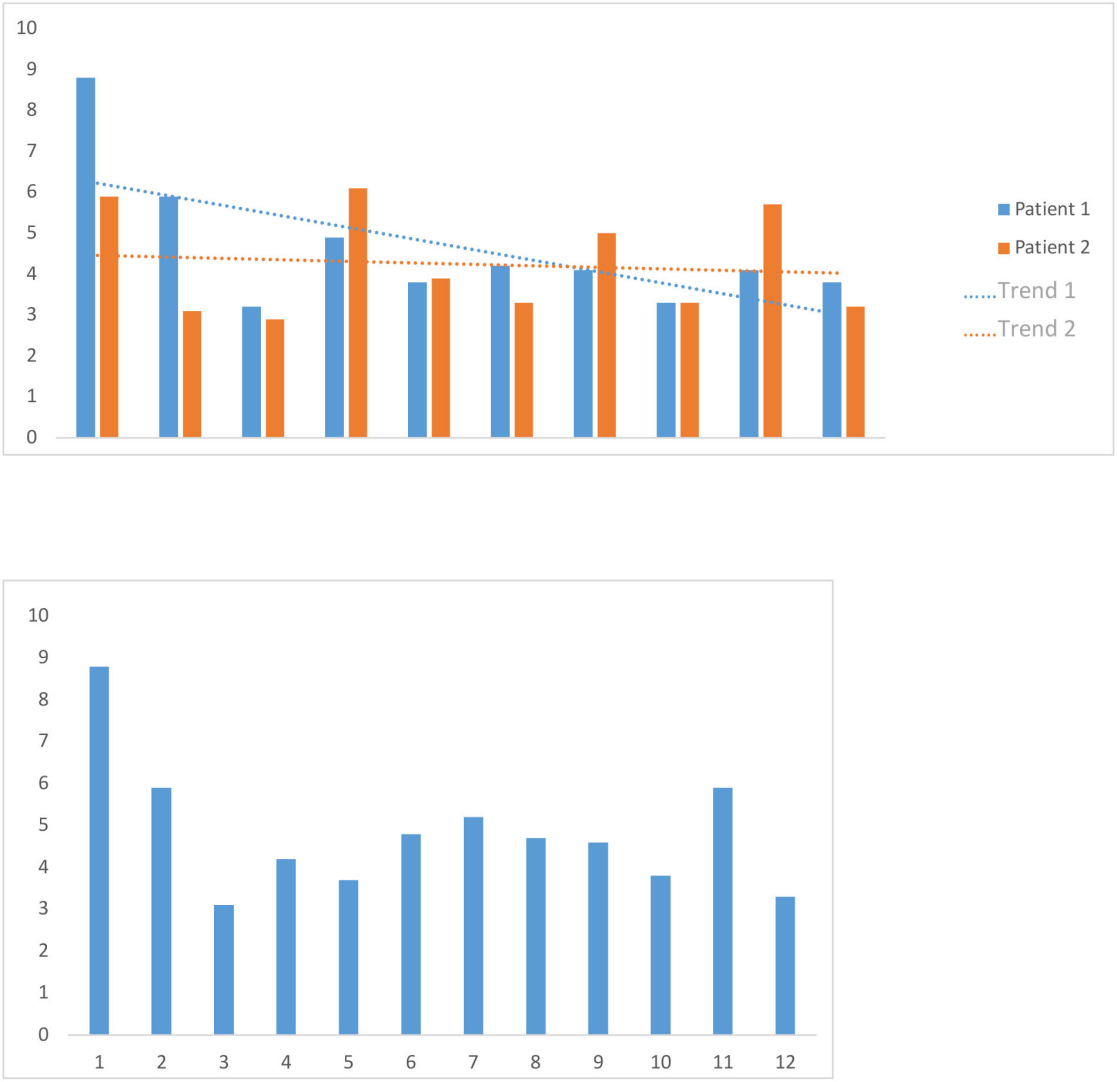
Image volume to the cerebral cortex volume. We calculated the image volume to the cerebral cortex volume of an average adult relevant to our study by applying a three-dimensional binary mask, which we obtained by increasing the contrast from an FDG-PET examination of a healthy adult patient's cortex.

The average duration of the interactive overlay of new records was 4.7 min, with a minimum of 3.0 min and a maximum of 8.7 min. The mean overlay duration of the records of two randomly selected patients was, for the first patient, 4.2 min, with a minimum of 2.8 min and a maximum of 6.2 min, and 4.6 min for the second patient, with a minimum of 3.3 min and a maximum of 8.7 min. Globally, in multiple overlays of the same records, the overlaying time shortens slightly the more overlay operations are carried out (Figure 5).

We timed the first overlay of 12 patients' data sets and used the mean as a benchmark for the first overlay of new data sets. We also timed 10 consecutive overlay attempts on the data sets of two randomly selected patients, then calculated the two mean values and examined whether there was a trend in them.

### Validation of the Vein Representation

**Figure 6:**
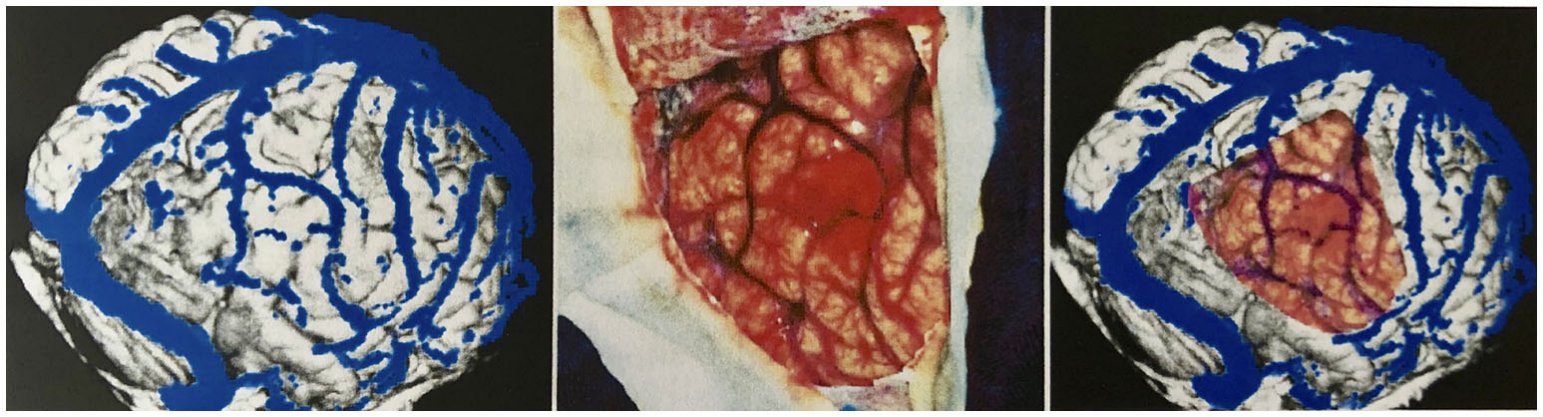
Validation of vein display. Transparent superimposition (**right**) of the reconstruction (**left**) and photograph of the surgical site (**middle**) for the validation of the vein display.

To measure the reliability of the reconstructions we made, we took intraoperative photographs of each patient's exposed cerebral cortex and of the vessels running on it. Based on these photographs, we then created views of our reconstructions from the same angle we took intraoperatively. The reconstruction and the photo were then aligned by rotating and scaling them in a commercially available image processing program (Adobe Photoshop 7.0) (Figure 6).

The brain shift (BS) phenomenon is the basis for a geometric comparison of the reconstruction and the photograph, down to the millimeter, as a mode for correlation. Therefore, we performed a quantitative comparison focusing on neurosurgically relevant veins. For this purpose, relevant veins were marked both in the reconstruction and in the photo. Since it is difficult to determine which vein is the stem and which the confluence vessel in junctions and forks, we decided to mark each subarea individually to facilitate the evaluation.

**Figure 7:**
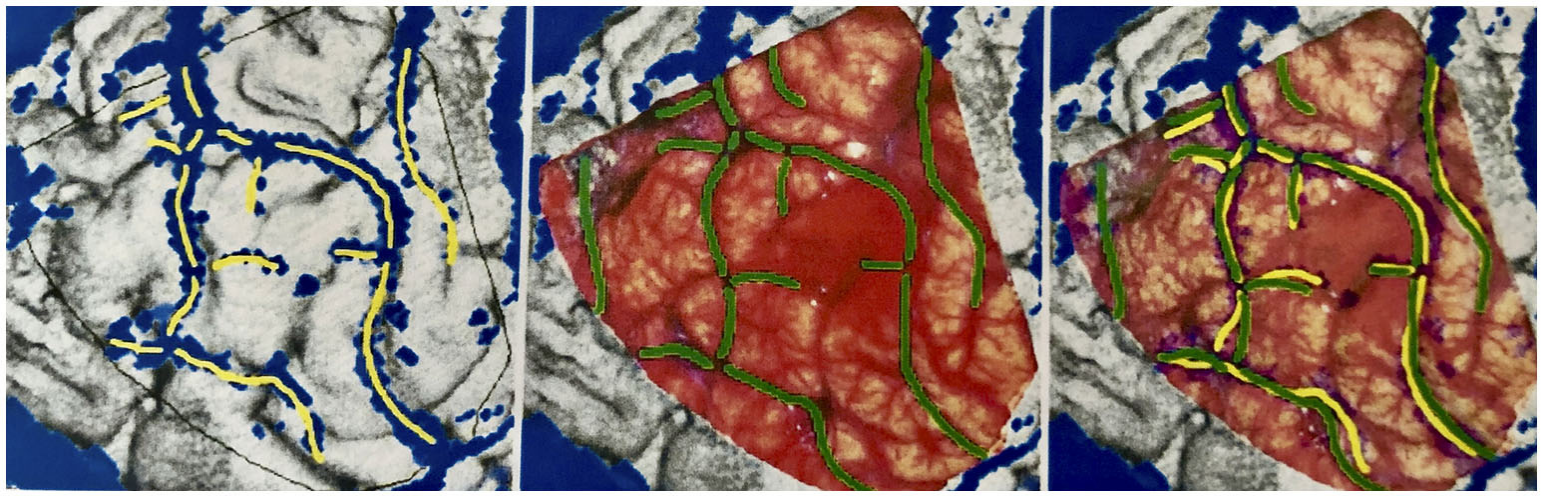
Marking the veins shown on different drawing levels and evaluation of the levels by superimposition. **Left**: In the reconstruction, vein sections identified as such are marked in yellow. **Middle**: In the surgical site photograph, important vein sections for the neurosurgeon are marked in green. **Right**: The yellow and green markings are displayed one above the other for evaluation.

**Figure 8:**
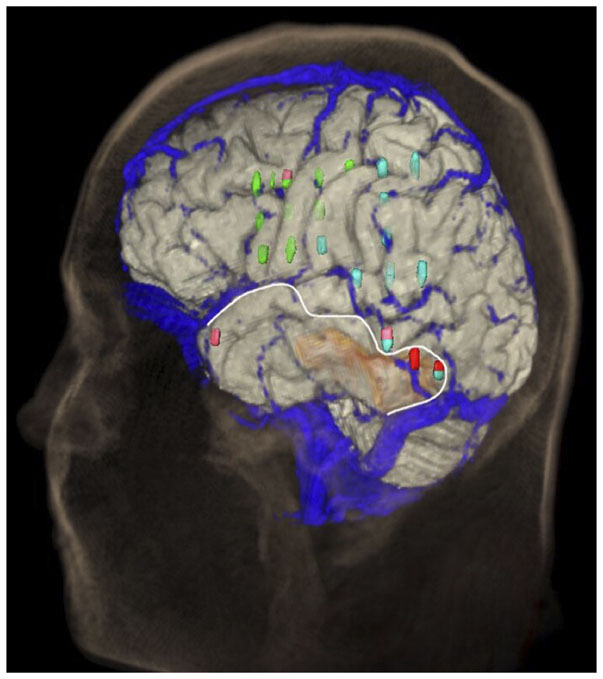
Distribution of the bridging veins and their junctions with the SSS. Anastomosing veins are highlighted in red. This illustration was made by Prof. Peter A. Winkler, based on the results of our anatomical considerations.

To mark all the structures we identified as veins in the reconstruction, we desaturated the photo and traced in yellow, on a separate layer, the veins' course. Similarly, the veins found in the photograph of the surgical site were marked in green on a new layer, in cooperation with the neurosurgeons who operated, as it was essential for us to mark all the vessels relevant for the surgical procedure. Fine lateral or end branches without great relevance to perioperative complications were not marked (Figures 7, 8).

The layers with the green and yellow markings were displayed simultaneously to assess the reconstruction's quality, and the results were evaluated using the scheme shown in Table 1. We classified each marked vein section as one of this scheme's five categories and recorded each category's frequency as a percentage. The vein sections marked in the same way in photograph and reconstruction were classified as Category 0; therefore, this category's frequency is a measure of our vein presentation method's reliability.

**Table 1:** Overview of veins categories.

**Category**	**Definition**	**Example**
0 (= MR +, Photo +)	Vein in photo and in reconstruction corresponds unambiguously (=true positive).	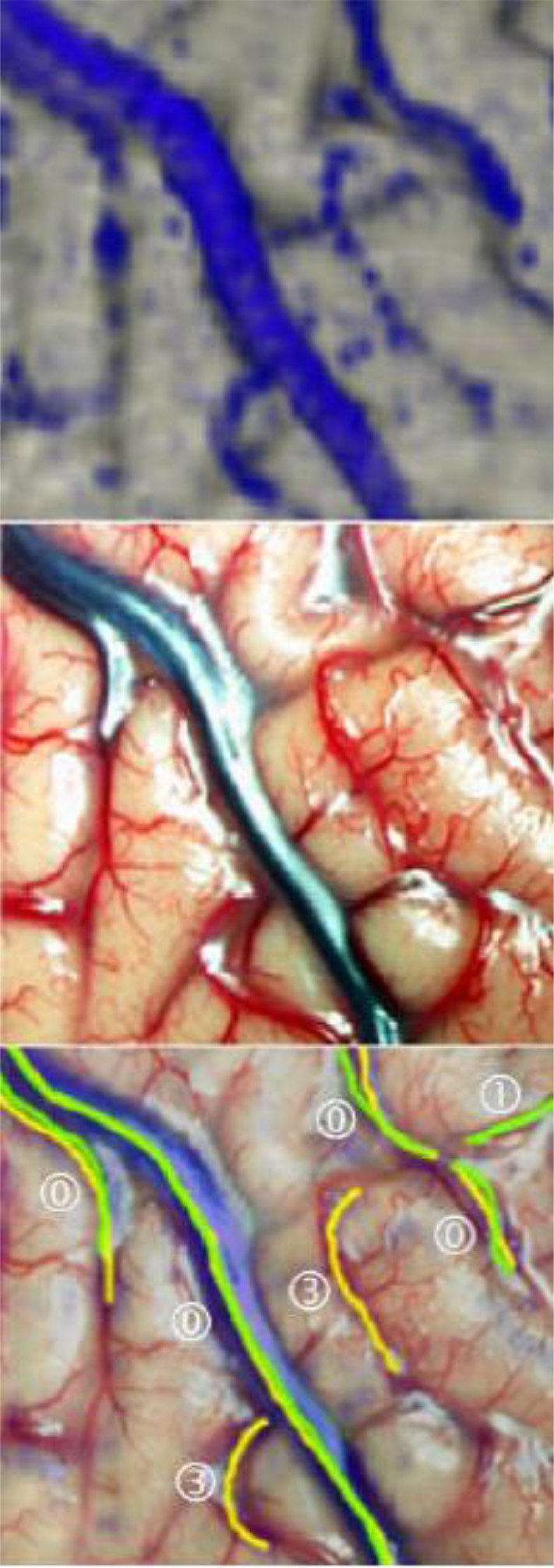
1 (= MR ϕ, Photo +)	Vein in photo without correspondence in reconstruction (=false negative).	
2 (= MR +, Foto ϕ)	Vein in reconstruction without correspondence in photo (=false positive).	
3 (= arterial)	Vein in reconstruction, represented as artery in photo (=false positive).	
4 (= not assessable)	Vein in reconstruction is not visible or assessable in photo.	

*Each colored vein has been classified in a category of this schema*.

*The right column shows an example of illustration, circled to the figure corresponding to the category in which we classified the vein*.

### Anatomical Examinations

In order to use the reconstructions created within the framework of this manuscript for the performance of anatomical observations, we created several images of each patient, showing the brain and the cortical vessels running on it from different fixed angles. The views we used are shown in Table 2.

**Table 2:** Overview of the anatomical analysis and the standardized views of reconstructions.

View 1: Perpendicular cranial view Reconstruction of bridging veins and of confluence angles	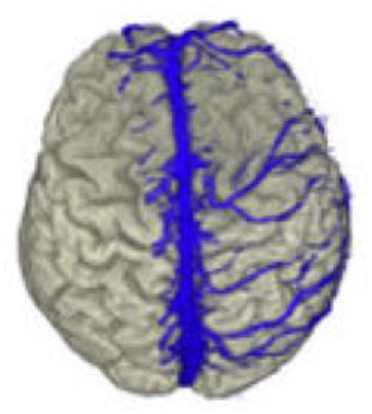	View 4: Lateral view, 30^°^ cranial Reconstruction of anastomotic and of bridging veins	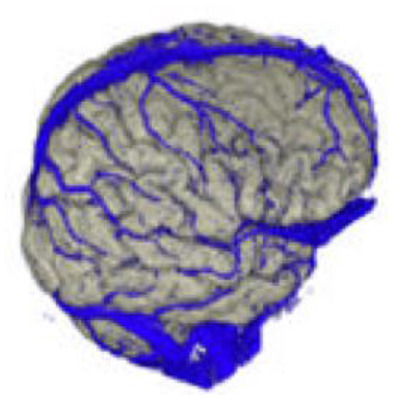
View 2: Top view, 40^°^ frontal Reconstruction of bridging veins and of confluence angles	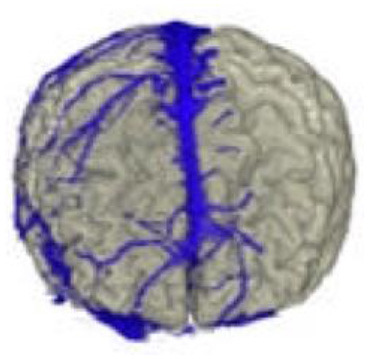	View 5: 30^°^ frontal, 30^°^ cranial Reconstruction of anastomotic and of bridging veins	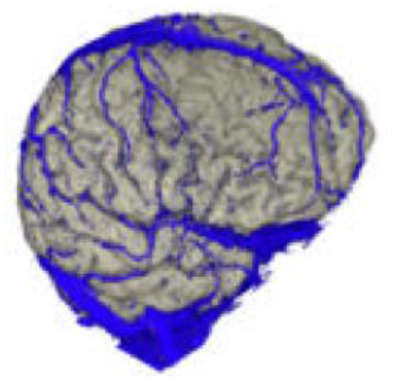
View 3: Top view, 40^°^ occipital Reconstruction of bridging veins and of confluence angles	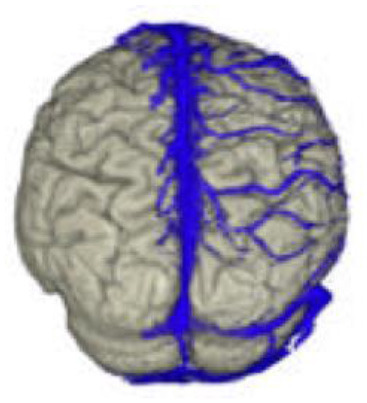	View 6: 30^°^ occipital, 30^°^ cranial Reconstruction of anastomotic and of bridging veins	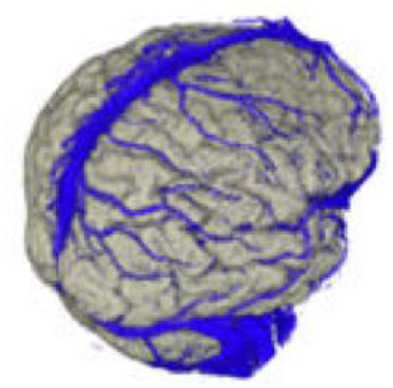

### Determination of the Number of Bridging Veins at the SSS

We examined 23 patients' reconstructions in the views 1–3 presented in Table 1 for the number of bridging veins entering the SSS. This procedure was performed separately for the left and right hemispheres, as well as for the frontal, middle and occipital thirds of the superior sagittal sinus. We summed the mean values for the individual sinus sections to obtain the average number of bridging veins on the corresponding side of the sinus.

### Determination of Drainage Types Frequency

We investigated the occurrence of large anastomotic veins in 19 of our patients and classified them according to the types of venous drainage of the lateral cerebral hemispheres presented by Erös et al. (1999). For this purpose, we used the views 4–6 of the reconstructions, presented in Table 1. In four patients, the arrangement of the layer stack of the MR angiography prevented an assessment of the venous anastomoses.

### Determination of the Bridging Veins' Confluence Angle at the SSS

In order to determine the bridging veins' confluence angle at the superior sagittal sinus from our reconstructions' views 1–3, it was necessary to think about the underlying type of imaging and the resulting geometric conditions (see Appendix).

We divided the bridging veins at the SSS into six different groups, based on the localization of their confluence: anterior frontal, medial frontal, posterior frontal, anterior parietal, posterior parietal, and occipital. As far as possible, we determined the confluence angles for two veins per group in each patient. We calculated 267 angles: 46 for the anterior frontal group, 45 for the medial frontal, 45 for the posterior frontal group, 44 for the anterior parietal, 46 for the posterior parietal, 41 for the occipital group. For each group, we calculated the mean and mean absolute deviation values. For the distance and angle measurements, we used Adobe Photoshop 7.0. Whenever possible, we compared and verified the anatomical considerations made on the reconstructions with the corresponding intraoperative photos.

## Results

### Confluence Angle of the Bridging Veins at the SSS

The veins draining the frontal lobes show approximately a right angle at the SSS level (Table 3). The values we measured for the bridging veins' mean confluence angle in the various sections of the SSS, and the mean absolute deviation (MAD) from these mean values as a measure of the values' range, are shown in Table 4.

**Table 3:** Average confluence angle of superficial cortical veins at the SSS level.

**Vein**	**Range**	**Average** **confluence angle**
V. frontopolaris	85°–150°	110°
V. frontalis anterior	55°–155°	110°
V. frontalis media	20°–160°	85°
V. frontalis posterior	15°–105°	65°
V. praecentralis	20°–80°	50°
V. centralis	10°–95°	45°
V. postcentralis	15°–90°	40°
V. parietalis anterior	0°–55°	25°
V. parietalis posterior	0°–32°	15°
V. occipitalis	0°–45°	10°

*The veins draining the frontal lobes show approximately a right angle at the SSS level*.

**Table 4:** Values-measurements for the bridging veins' mean confluence angle in the various sections of the SSS, and the mean absolute deviation from these mean values (MAD) as a measure of the range of the values.

	**Anterior frontal**	**Medial frontal**	**Posterior frontal**	**Anterior parietal**	**Posterior parietal**	**Occipital**
Mean	103.5°	85.2°	69.5°	61.1°	47.0°	39.3°
MAD	14.5°	10.3°	15.3°	11.9°	11.2°	7.9°

### Results of the Correlation

In our reconstructions, we identified a total of 158 vein sections. The intraoperative photographs showed 161 relevant veins. The total number of veins categorized by us was 179. In 140 cases, we found a match (Category 0 in Figure 1), which corresponds to 78.2%. Twenty-one patients had veins visible in the photograph that were not shown in our reconstructions (Category 1—11.7%). In eight cases, veins identified in the reconstructions were not visible in the photographs (Category 2—4.5%). In four patients, structures identified as veins in the reconstruction were recognized intraoperatively as arteries (Category 3—2.2%). Finally, in six cases (3.4%), veins identified in the reconstructions could not be assessed in the surgical site (Category 4), as they were covered by cotton wool, already coagulated, or resected together with the surrounding tissue before we took the photograph.

### Geometric Error of the Interactive Co-registration

In the three patients, the geometric deviation from the 10 overlay attempts' gold standard was 0.90 mm in the first patient, 1.20 mm in the second patient, and 0.58 mm in the third patient. The intraobserver variability of the interactive co-registration procedure used by us, calculated from these three values, is 0.89 mm (Table 5A).

**Table 5A:** Determination of intra-observer variability.

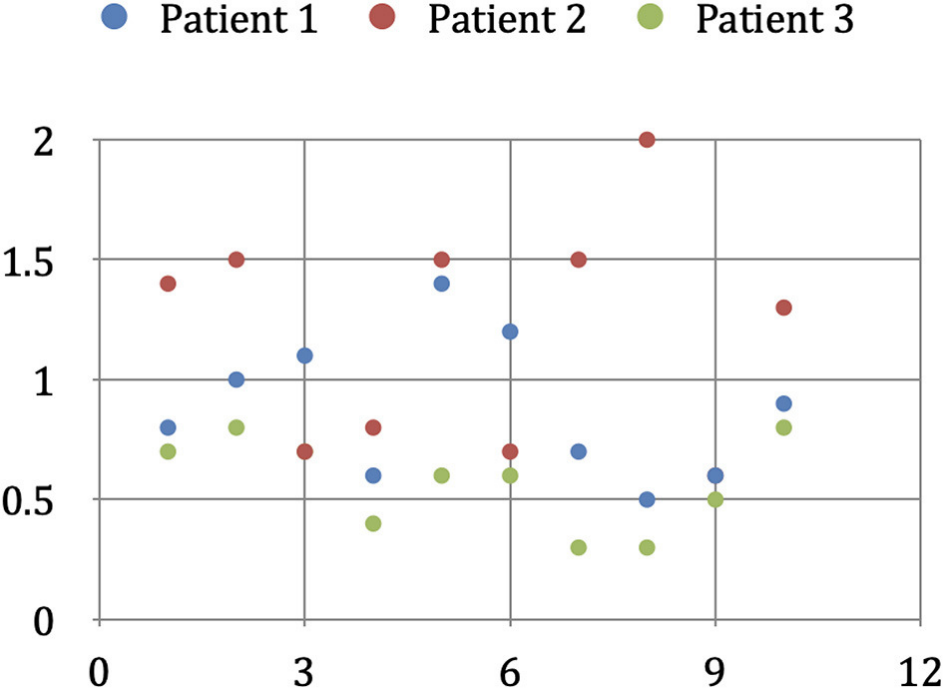

*The deviation from the gold standard of the individual laying tests for 10 attempts in 10 patients is graphically illustrated*.

In our interobserver variability studies, the two experienced users (users 1 and 2) achieved average geometric deviations of 0.71 and 1.32 mm from the gold standard. The less experienced user (user 3) achieved a value of 0.61 mm, while the inexperienced users (users 4 and 5) achieved average deviations of 0.57 and 0.87 mm from the gold standard. The average deviation of each user was 0.82 mm, which is the interobserver variability value of our method (Table 5B).

**Table 5B:** Determination of the inter-observer variability.

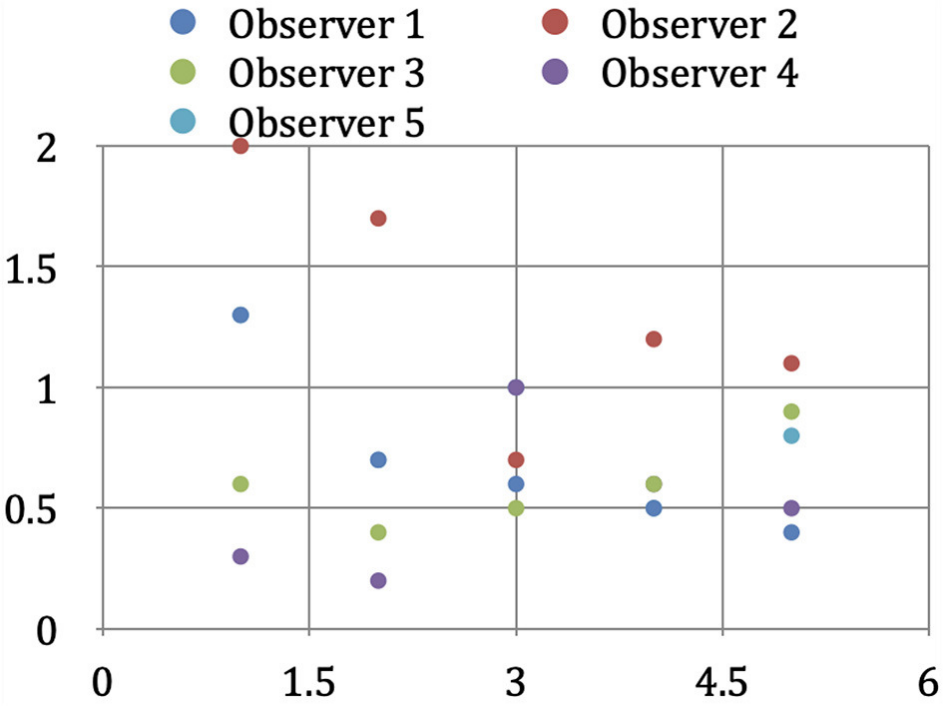

*The deviation from the gold standard of the individual laying tests for each attempt of five users with different experience is graphically illustrated*.

### Results of the Anatomical Considerations

#### Number of Bridging Veins

We found an average of 10.9 bridging veins on the left and 10.5 bridging veins on the right of the superior sagittal sinus during the examination of the 23 reconstructions in our study. We observed an average of:

- 4.3 bridging veins on the left and 4.1 on the right of the frontal third of the sinus;
- 4.4 bridging veins on the left and 4.5 on the right of the middle third of the sinus;
- 2.3 bridging veins on the left and 2.0 on the right of the occipital third of the sinus.

#### Frequency of Drainage Types

**Figure 9:**
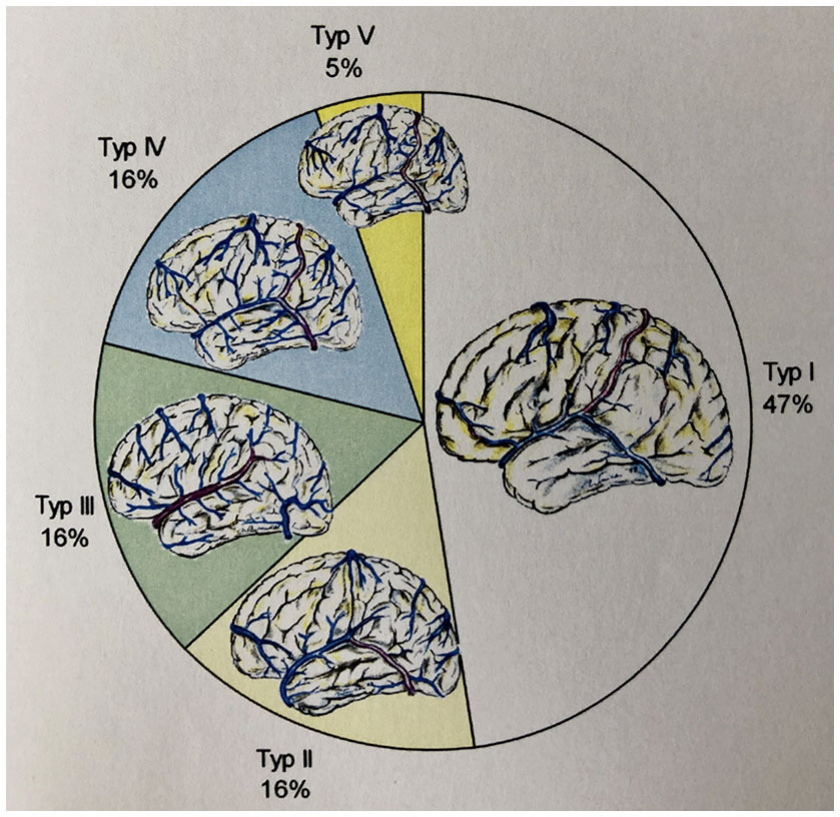
Frequency of the different drainage types (according to Erös et al.) in our study's patient population.

We examined 19 reconstructions of the cortical venous anatomy for the drainage type using the classification by Erös et al. We found a Type I configuration in nine cases. We assigned three patients each to Type II, III, and IV, and one patient to Type V cortical venous drainage (Figure 9 and Table 6).

**Table 6:** Distribution of the found drainage types (according to Erös et al.) in the reconstructions created by us.

**Drainage type**	**Amount (total)**	**Side**	
		**R**	**L**
Type I	9	6	3
Type II	3	1	2
Type III	3	1	2
Type IV	3	1	2
Type V	1	1	–

#### Results: Adjustment of the Threshold Value

If the threshold value is set too high, the veins are separated clearly from the brain parenchyma in the reconstruction, but they tend to present themselves discontinuous and broken, and smaller veins and branches do not appear at all, thus increasing the number of false negatives. If, on the other hand, the threshold value is set too low, the main stems are continuous, and the veins are more finely branched, but it is often difficult to distinguish the vessels from the blue “background noise” of the brain parenchyma, and the veins from artifacts. In this case, one would experience an increase in false positives.

## Discussion

Preoperative knowledge of the cerebral cortex anatomy, and of the veins running on it, helps the surgeons plan and execute a neurosurgical procedure and avoid complications. Several methods have been developed, in recent years, to visualize the course of the cerebral vessels, often limiting the visualization only to the veins by appropriate methods (conventional angiography, CT angiography, MR angiography) or with imaging reconstruction (Di Chiro, 1962; Kaplan and Browder, 1974; Katada, 1990; Lüders, 1992; Imai et al., 1996; Kikinis et al., 1996; Nakajima et al., 1997; Hawkes, 1998; Tsuchiya et al., 1998, 1999a,b, 2002; Kettenbach et al., 1999; Kubota et al., 2001; Nabavi et al., 2001; Kaminogo et al., 2002; Murphy et al., 2004). To reconstruct the cortical anatomy of the cerebral hemispheres, we used a strongly T1-weighted MPRAGE sequence (Padget, 1956; Sugita et al., 1982; Lanzieri et al., 1987; Liang et al., 2001). These sequences have been used for years (Tsutsumi et al., 1991; Weber and Ivanovic, 1994; Guppy et al., 1997).

Digital Subtraction Angiography (DSA) was the gold standard for intracranial vascular imaging until a few years ago, but it is an invasive procedure with a non-negligible rate of complications (von Lanz and Wachsmuth, 1985; Laub, 1994; West et al., 1997). The method of choice for presenting and evaluating cerebral veins has been described in the literature (Huber, 1982; Lee et al., 1997). The bridging veins and the large cortical anastomotic veins examined in this study can be visualized with CT angiography in 76–97% of cases. Before beginning our study and including patients, we decided to use MR angiography to visualize the cortical veins and examined volunteers with different MRA sequences and contrast agent dosages. We first tried to image the cortical veins using contrast-enhanced MPRAGE sequences, which was successful thanks to the independence of layer orientation and flow velocity (Philips Medical Systems, 1996; Liang et al., 2001). However, it turned out that the smaller superficial veins were only recognizable after the administration of higher doses of paramagnetic contrast medium. The resulting uptake of contrast medium by the meninges posed a problem in the differentiation of the cortical veins from the meninges, significantly limiting the cortical surface's virtual segmentation. Therefore, we turned to flux-sensitive MRA sequences. Slow blood flow in the smaller cortical veins limits their representation in the MRA (Cline et al., 1991; Gandhe et al., 1994; Pant et al., 1997; Mamata et al., 1999; Reichenbach et al., 2000; Wilms et al., 2001). Also, changes in flow direction and velocity, caused by various pathological factors (tumor, edema), can make the representation of venous structures more difficult or impossible (Noachtar et al., 2003). For example, the time-of-flight method might be inadequate to distinguish vessels from stationary tissue at low flow velocities (Bartels and van Overbeeke, 1997). The situation is similar in phase-contrast MRA, where the motion-induced phase shifts may not be detected in slow-flowing blood (Bartels and van Overbeeke, 1997). The loss of signal in vessels that run mostly parallel to the layer orientation can be misread as occlusion or absence of blood vessels (Bartels and van Overbeeke, 1997). For this reason, we oriented the layer stack at an angle of at least 10° to the SSS's course to obtain a good representation of the sinus. Furthermore, we tried to orient the layers in the relevant areas as perpendicular to the cortical surface as possible, to represent the veins better and prevent saturation of the signal of smaller vessels in this region.

MRA may be improved by developing MRIs that work with higher magnetic field strengths–MRAs in the range of 3 Tesla can both significantly improve the spatial resolution and shorten the duration of the examination (Gandhe et al., 1994).

We are aware that there is still potential for optimizing the MR angiography sequence we used. The 2D-TOF sequence does not always detect smaller veins, especially in the temporal region. The maximum of 64 layers is also an obstacle, as the sagittal layers—with a maximum thickness of 2 mm and width of 12.8 cm—do not allow simultaneous imaging of both hemispheres' veins. A more suitable sequence could probably also reduce, if not altogether avoid, the use of paramagnetic contrast agents. A shorter examination time would also be desirable to minimize movement artifacts and stress on the patient.

### Registration Method

The registration method we use is interactive and retrospective (Chan and Thompson, 1984; Wenz et al., 1994; Liang et al., 2001). Despite the growing applications of automatic registration, the interactive procedures show a comparable or better accuracy. With interactive image superimposition, the operator can limit the result.

Similarly to a study by Pfluger et al., in our investigations no significant difference was noted between people knowledgeable in both clinical anatomy and three-dimensional image superimposition and laypersons without background knowledge in the evaluation of superimpositions (Wong et al., 1997). The lay group's expected error was also below the value of automatic procedures, such as Wood's algorithm (Woods et al., 1992; Bosmans and Marchal, 1996; Wong et al., 1997). With an average duration of 4.7 min for the primary and secondary data sets' co-registration and a rendering time usually of <30 min, our procedure can be easily integrated into clinical routine Globally, in multiple overlays of the same records, the overlaying time shortens slightly the more overlay operations are carried out.

### Validation of the Vein Representation

A reconstruction only has clinical value if it reflects, as reliably as possible, the real anatomical conditions, and intraoperative comparison with the patient's real situs is the best way to compare the two. By marking the veins independently on several layers and subsequently evaluating the marked sections in cooperation with the neurosurgeons performing the procedure, we obtained a concrete quantitative evaluation of the procedure. With a positive correlation of 78.2%, our method for imaging superficial cortical veins and cortical gyral anatomy proved to be a reliable aid in planning and performing neurosurgical interventions. In 11.7% of cases, we could not visualize the relevant veins preoperatively. These were mostly smaller cortical veins, often in the temporal region. This obstacle is an incentive to optimize further the MR angiography sequence we use. The ratio of false-positive veins (4.5%) is associated with the topic of “suitable threshold value.” Identifying cortical arterial vessels as veins, which happened in 2.2% of the marked sections, is unavoidable when using flow-sensitive MRA sequences.

### Anatomical Considerations

Every imaging reconstruction provides an incomplete image of reality. Casey et al. reported an average of 6.2 bridging veins to the SSS on each side, while the mean value in our studies was significantly higher, with 10.5 and 10.9 veins per side (Casey et al., 1998). A different sensitivity and specificity of the methods employed, and/or a different interpretation of the reconstructions, could explain this discrepancy. As we were present during the operations and photographed the surgical site, we were able to compare the veins reconstructed with MR imaging to the real ones. We confirmed the results of our anatomical observations in all cases.

The method we have developed for measuring confluence angles has proven to be extremely robust and easy to use in practice. Comparative measurements of the same angles in different images showed only minor deviations in the range of ±5°.

## Limits and Strengths of the Study

The technology used in this study could appear dated or old fashioned and—as it requires manual intervention—even difficult to use in the clinical practice, compared with other medical imaging software currently in use in our operating rooms.

However, this limit is not necessarily a weakness. The Brain Shift is particularly important in the navigation systems, which are usually focused on preoperative imaging procedures. Consequently, the intraoperative shift always happens, especially on the brain surface, distorting the superficial venous patterns and causing spatial variations.

In this context, the method for visualizing superficial veins we presented is robust, as the veins on the cortical surface experience the brain shift phenomenon and therefore are not limited to the cortical landmarks or are only minimally displaced.

Furthermore, at present, the study represents the most extensive and scholarly work on the subject, including mathematical and geometrical calculations of the veins flowing into the superior sagittal sinus, which have never been presented before.

## Conclusion

Three-dimensional reconstructions of the cortical anatomy and its superficial veins help plan and optimize neurosurgical procedures and minimize complications. Our method for visualizing superficial veins helps manage brain shift (BS), as the veins on the cortical surface shift together with the underlying brain tissue and therefore do not—or only minimally—change their relative location to the cortical landmarks. We developed a method for the combined representation of the cortical anatomy and the superficial cerebral veins that could represent a valuable tool, and a possible adjunct in the neuronavigation armamentarium, in planning and performing neurosurgical interventions. Due to the good correlation with the existing surgical site, it should be validated on a larger cohort or in a multicentric study.

## Data Availability Statement

The original contributions presented in the study are included in the article/supplementary materials, further inquiries can be directed to the corresponding author/s.

## Ethics Statement

Ethical review and approval was not required for the study on human participants in accordance with the local legislation and institutional requirements. Written informed consent for participation was not required for this study in accordance with the national legislation and the institutional requirements. Written informed consent was not obtained from the individual(s) for the publication of any potentially identifiable images or data included in this article.

## Author Contributions

All authors listed have made a substantial, direct and intellectual contribution to the work, and approved it for publication.

## Conflict of Interest

The authors declare that the research was conducted in the absence of any commercial or financial relationships that could be construed as a potential conflict of interest.
