# Supplementary material for: Importance of Veins for Neurosurgery as Landmarks Against Brain Shifting Phenomenon: An Anatomical and 3D-MPRAGE MR Reconstruction of Superficial Cortical Veins
Source: Front Neuroanat. 2020 Dec 15;14:596167. doi: 10.3389/fnana.2020.596167 (PMC7771049; doi:10.3389/fnana.2020.596167)
Supplement: Supplementary file 1 [file Data_Sheet_1.pdf]

Appendix

While the number of cortical veins, their courses, and the anastomoses they form can be seen at a glance, calculating the angle between the bridging veins and the SSS represents a small mathematical challenge.

Like any 2D image of a three-dimensional body, the images we create with the VoxelQ workstation may involve some optical distortion; therefore, it is not possible to measure angles directly on these images. However, knowing the imaging methods used, and assuming some simplification, it is possible to calculate these angles using geometric considerations.

The workstation displays the images generated by us as a normal projection, which means that the lines connecting original and image points run parallel to each other and perpendicular to the image plane (see Fig. A.1). To have at least one representation of each junction between a bridging vein and the superior sagittal sinus from frontal to occipital, we used the views 1 to 3 presented in *Anatomical examinations* to calculate the angles. View 1 shows the brain vertically from above – the image plane runs parallel to the horizontal plane. View 2 is at an angle of 40° to the horizontal and shows the brain from the front. View 3 is at an angle of 40° to the horizontal and shows the brain from the occipital plane. All three image planes are also orthogonal to the sagittal plane (see Fig. A.2).

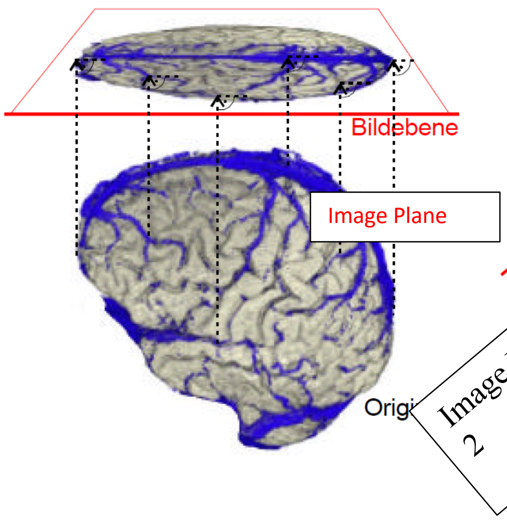

Fig. A.1: Schematic representation of a normal projection of a 3D body onto a 2D image plane.

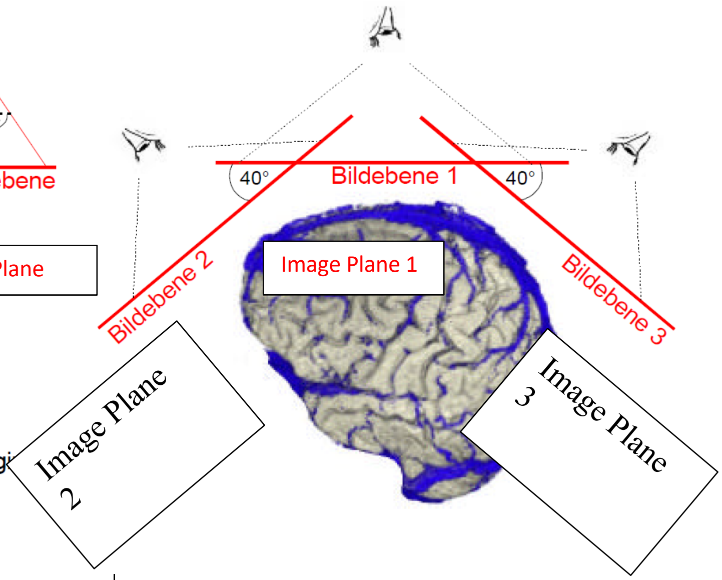

Fig. A.2: Representation of the projections we have created for angle calculation, and their associated views.

To keep the effort to calculate the confluence angles within a manageable mathematical framework, we made some simplifying assumptions. For example, we assumed that the brain surface from the vertex to the level of the Sylvian Fissure has the shape of a sphere, which often comes very close to the natural anatomical conditions. Furthermore, the superior sagittal sinus in our model runs equatorially from the most frontal (Fr), through the highest (H), to the most occipital point (Oc) (see Fig. A.3).

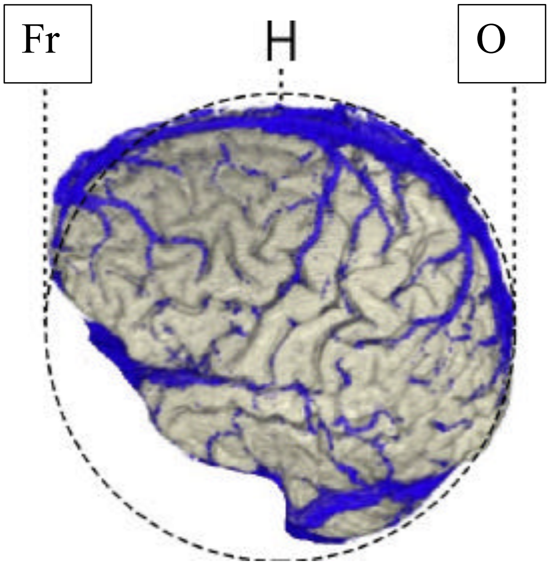

Fig. A.3: Illustration of the simplifying assumptions.

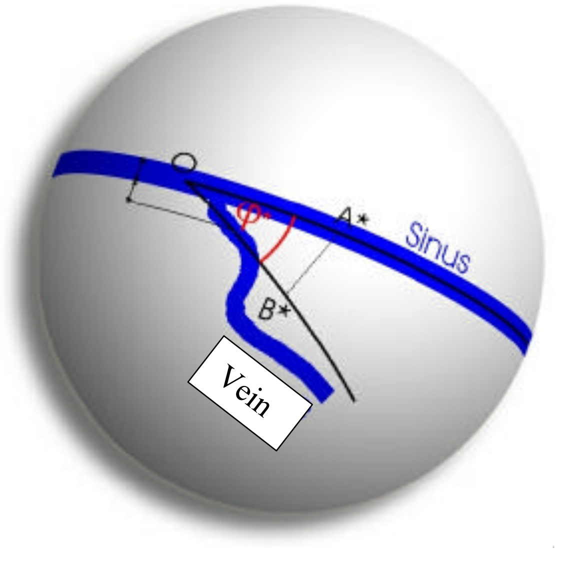

Fig. A.4: Selection of the triangle $A^*\overset{\Delta}{;}OB^*$, on the surface of the sphere, which contains the angle φ.*

To determine a bridging vein's confluence angle, we select a triangle $A^*\overset{\Delta}{;}OB^*$ on the sphere's surface. To do this, we place a tangent at a distance of one sine width through the bridging vein in question. This straight line intersects the sine perpendicularly at point O.

The angle φ* included in the vertex O is the bridging vein's corresponding real confluence angle (see Fig. A.4). Point A* is located on the straight line running along the middle of the sinus, at any distance from O. Point B* is located, at any distance from O, on the straight line running through the vein. Points A* and B* are selected so that the resulting confluence angle is acute ($\varphi^* < 90°$).

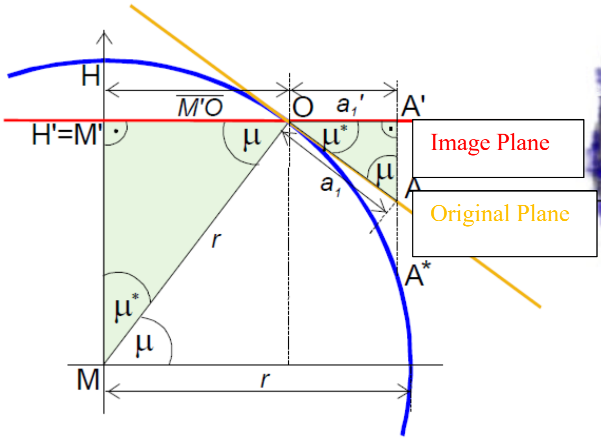

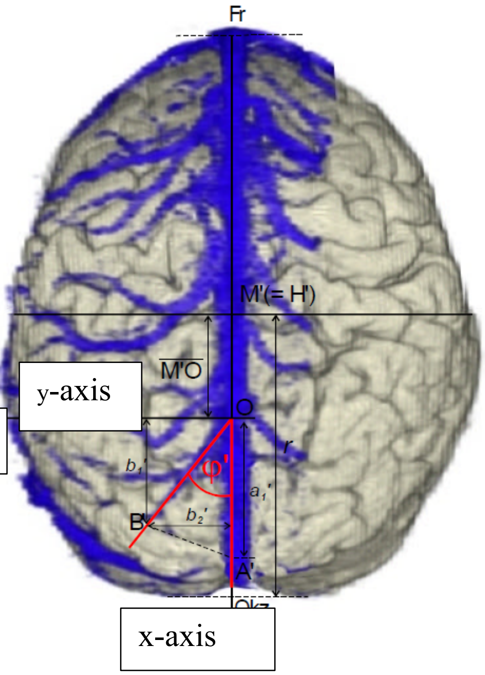

Fig. A.5: Section in the sagittal plane and passing through the SSS.	*Fig. A.6: Determination of the angle φ' on the normal projection of φ* onto View 1.*

The following considerations concern only View 1, which is parallel to the horizontal plane. Two image planes are passed through point O: a plane on which the reconstruction is projected using parallel projection (Image Plane), and a tangential plane that touches the sphere surface at point O (Original Plane) – see Fig. A.5. The intersection points of the tangent plane with the rays of the normal projection, which map the points A* and B* onto the Image Plane, are designated as A and B (Original Plane). The rays of the normal projection are then projected onto the Image Plane. Thus the spherical triangle A*Δ;OB*, whose sides are arcs, is transformed into a triangle AΔ;OB on the tangential plane, which simplifies further geometrical considerations. We call A′Δ;OB′ the triangle on the parallel projection plane, and the tangential plane at point O to the sphere surface the original plane (see Fig. 9.5). The triangle AΔ;OB is projected onto the Image Plane by normal projection;

in this way, the image triangle $\overset{\Delta}{A'};OB'$ is created (see Fig. A.6). In order to illustrate the following calculations, a coordinate system is placed onto each of the planes, where the x-axis follows either the sine or the tangential image of the sine (x_O-axis or x_B-axis). The vertex O is the origin, and the y-axis intersects both planes (Fig. A.7).

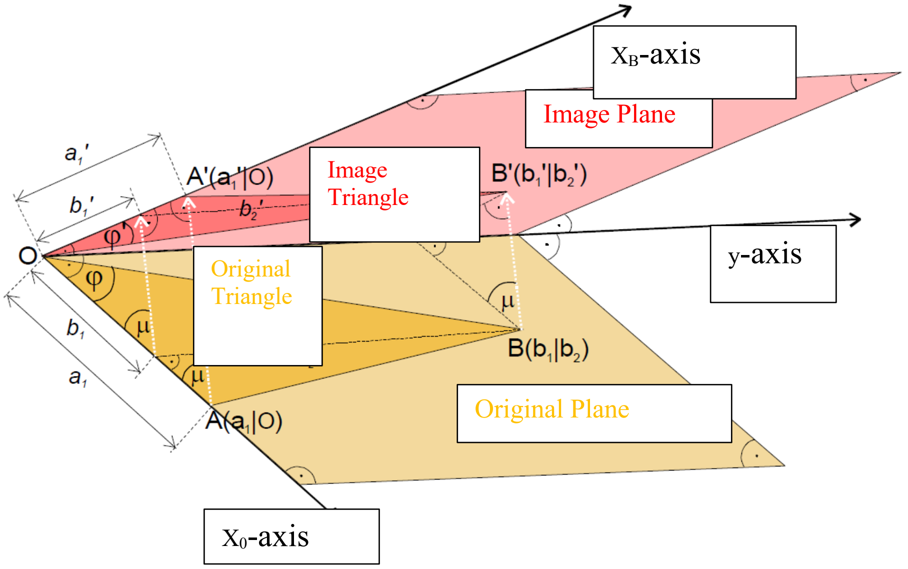

Fig. A.7: Diagram of the mapping of $\overset{\Delta}{A'};OB'$ from $\overset{\Delta}{A};OB$. The rays of the normal projection are shown in white.

The angle μ* corresponds to the angle between the Image Plane and the Original Plane (see Fig. A.5), and varies depending on the distance of the vertex O from point M′, which is the projection of the center of the sphere M onto the Image Plane. The angle μ* is calculated from μ, as μ* = 90°-μ. The angle μ corresponds to the angle of the“geographical latitude” of point O on the sphere if H is considered the north pole. To calculate the confluence angle φ, we measure the angle φ′, the

distance lengths a_1', b_1' and b_2', as well as the radius r and the distance $\overline{\quad}$;M'O in an image created by us after selecting the triangle A$'$ Δ;OB$'$.

According to Fig. A.7:

$$a_1' = a_1 \cdot sin\mu$$

$$b_1' = b_1 \cdot sin\mu$$

$$a_2' = a_2 = 0$$

$$b_2' = b_2$$

Furthermore, μ is determined by (see Fig. A.5):

$$\cos \mu = M' \frac{O}{r}$$

If one transforms the Original and the Image Planes along the y-axis so that both planes lie on one plane (x_O- and x_B-axis point in opposite directions), one can read and evaluate further geometric correlations (see Fig. A.8).

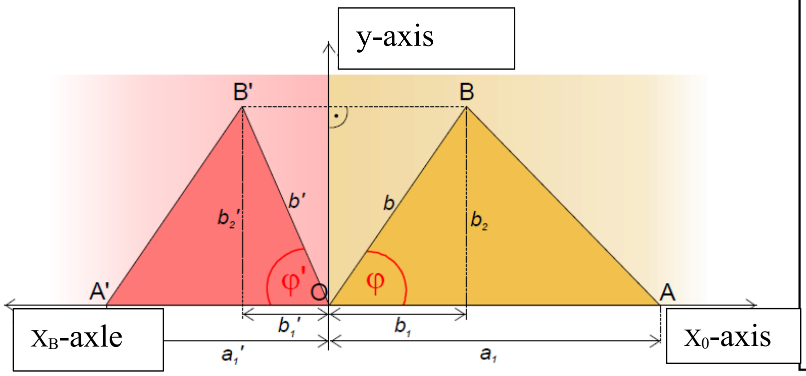

$$\cos \varphi = \frac{b_1}{b}$$

$$\cos \varphi = \frac{b_1}{\sqrt{b_1^{\,2} + b_2^{\,2}}}$$

$$\cos \varphi = \frac{(b_1' : \sin \mu)}{\sqrt{(b_1' : \sin \mu)^2 + b_2'^2}}$$

$$\cos \varphi = \frac{b_1'}{\sqrt{b_1'^2 + (b_2' \cdot \sin \mu)^2}}$$

Fig. A.8: View of the Image and the Original Planes transformed onto a single plane.

As can be seen from the formulas at the top right, it is possible to calculate the confluence angle from the quantities measured in our illustration and the angle μ calculated from them.

For the calculation of cosφ, no information about the position of point A′ is required, i.e., the fact that it lies on the sine is sufficient. In this respect, it seems evident that only part of the information is required for the position of point B′. This is to be confirmed by subsequent transformations.

It applies:

$$\cos\varphi = \frac{b_1'}{\sqrt{b_1'^2+(b_2'\cdot\sin\mu)^2}}$$ with $b_1' = b' \cdot \cos\varphi'$ and $b_2' = b' \cdot \sin\varphi'$

$$\cos\varphi = \frac{(b'\cdot\cos\varphi')}{\sqrt{(b'\cdot\cos\varphi')^2+(b'\cdot\sin\varphi'\cdot\sin\mu)^2}}$$

$$\cos\varphi = \frac{\cos\varphi'}{\sqrt{\cos^2\varphi'+\sin^2\varphi'\cdot\sin^2\mu}}$$

$$\cos\varphi = \frac{1}{\sqrt{1+\tan^2\varphi'\cdot\sin^2\mu}}$$

Since this result is also independent of b_1', b_2', and b', the distance of point B′ from O is also arbitrary. Thus, the calculation of the confluence angle can be significantly simplified. It is sufficient to determine the radius of the sphere, the distance of the vertex from the image of the center of the sphere, and the angle φ' in order to calculate the angle φ. In the concrete case, we measure the sphere's diameter from the frontal pole to the occipital pole, halve it, and thus obtain the center point M′ and the radius r. Then we determine the distance from M′ to the vertex O and measure the angle φ'. The confluence angle φ is then calculated as follows:

$$\varphi = \cos^{-1}\left(\frac{1}{\sqrt{1+\tan^{2}\varphi'\cdot\left(1-\left(\frac{\overline{M'O}}{r}\right)^{2}\right)}}\right)$$

In the following, the considerations for View 1 are to be extended to any view.

The image plane is no longer necessarily parallel to the horizontal but forms an angle ψ to it. The angle ξ is the angle between the image plane and the original plane.

As in the previous observations, instead of the triangle $A^{*}\overset{\Delta}{;}OB^{*}$, the triangle $A\overset{\Delta}{;}OB$ is observed on the tangential plane, at the apex O of the confluence angle. The Original Plane or Image Plane is identical to the x_O:y plane or x_B:y plane, where the x_O axis and the x_B axis follow the sine or image of the same.

Same as the previous calculations, the triangle $A\overset{\Delta}{;}OB$ has the corner point coordinates O(0;0), A(a_1;0), B(b_1;b_2), and the triangle $A'\overset{\Delta}{;}OB'$ the corner point coordinates O(0;0), A′ ($a_1'; 0$), B′ ($b_1'; b_2'$). The confluence angle φ is mapped to φ′.

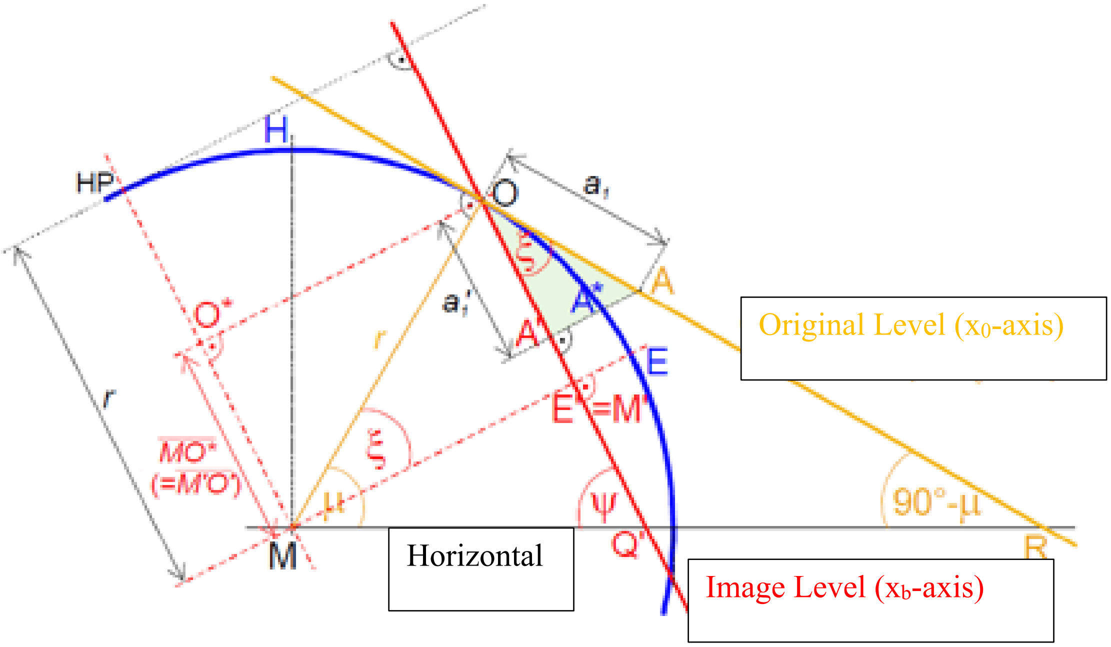

Fig. A.9: Section passing through the SSS. The Image Plane lies at any angle ψ to *the horizontal and is perpendicular to the sagittal plane.*

From the triangle A$\overset{\Delta}{}$;OA▯ in Fig. A.9, it follows:

$$a_1' = a_1 \cdot cos\xi$$

In triangle Q▯$\overset{\Delta}{}$;OR it applies:

$$\xi + (180° - \psi) + (90° - \mu) = 180°$$

$$\Rightarrow \xi = \psi + \mu - 90°$$

It therefore applies:

$$a_1' = a_1 \cdot \cos[(\psi + \mu) - 90°]$$

$$a_1' = a_1 \cdot [\cos(\psi + \mu) \cdot \cos 90° + \sin(\psi + \mu) \cdot \sin 90°]$$

$$a_1' = a_1 \cdot [\cos(\psi + \mu) \cdot 0 + \sin(\psi + \mu) \cdot 1$$

$$a_1' = a_1 \cdot \sin(\psi + \mu)$$

The same applies to this and to the considerations of the figure level 1:

$$b_1' = b_1 \cdot \sin(\psi + \mu)$$

$$b_2' = b_2$$

If the Original and the Image Planes are transformed along the y-axis, so that both planes lie on one plane (as for View 1 – Fig. A.8), it applies the same as before:

$$b_1' = b' \cdot cos\phi'$$

$$b_2' = b' \cdot cos\phi'$$

The following relationships can be derived from this:

$$\cos\varphi = \frac{b_1}{\sqrt{b_1^{2} + b_2^{2}}} = \frac{\dfrac{b_1'}{\sin(\psi+\mu)}}{\sqrt{\left(\dfrac{b_1'^{2}}{\sin^{2}(\psi+\mu)} + b_2'^{2}\right)}} = \frac{b_1'}{\sqrt{b_1'^{2} + b_2'^{2}\cdot\sin^{2}(\psi+\mu)}}$$

$$\cos\varphi = \frac{b'\cdot\cos\varphi'}{\sqrt{b'^{2}\cdot\cos^{2}\varphi' + b'^{2}\cdot\sin^{2}\varphi'\cdot\sin^{2}(\psi+\mu)}} = \frac{\cos\varphi'}{\sqrt{\cos^{2}\varphi' + \sin^{2}\varphi'\cdot\sin^{2}(\psi+\mu)}}$$

$$\cos\varphi = \frac{1}{\sqrt{1+\tan^{2}\varphi'\cdot\sin^{2}(\psi+\mu)}}$$

From this general formula for any image plane, the formula for the particular case of View 1, parallel to the horizontal ($\psi = 0°$) can be derived:

$$\xi = \mu^* = 90 - \mu$$

$$\Rightarrow \cos\xi = \cos(90° - \mu) = \sin\mu$$

$$\Rightarrow \cos\varphi = \frac{1}{\sqrt{1+\tan^{2}\varphi'\cdot\sin^{2}\mu}}$$

Instead of calculating μ, we recommend substituting it with $\xi = \psi + \mu - 90°$:

$$\psi + \mu = \xi + 90°$$

$$\Rightarrow \sin(\psi + \mu) = \sin(\xi + 90°)$$

$$\Rightarrow \sin(\psi + \mu) = \cos \xi$$

Thus:

$$\cos \varphi = \frac{1}{\sqrt{1 + \tan^2 \varphi' \cdot \cos^2 \xi}}$$

Which results in:

$$\sin \xi = \frac{\overline{M'O}}{r}$$

$$\Rightarrow \quad \xi = \sin^{-1}\left(\frac{\overline{M'O}}{r}\right)$$

The determination of r is problematic when choosing any image plane, since the brain's surface, as mentioned above, approaches a spherical shape only in the area above the Sylvian Fissure. Therefore, we used the value for the sphere's radius r – which we had previously determined from View 1, belonging to Image Plane 1 – to determine the confluence angle in the views whose associated image plane was not in the horizontal plane. Then, to determine the distance $\overline{M'O}$, we determined the point E, at distance r from the sphere's visible upper horizon (HP) (see Fig. A.9).

Remarkably, it follows from the final result that for the calculation of φ only the image angle φ[illegible] and the angle ξ between the Original Plane and the Image Plane are needed. This also means that there is no error if you look at the triangle $\overset{\Delta}{AOB}$ on the tangential plane instead of the triangle $\overset{\Delta}{A^*OB^*}$ ($\varphi^* = \varphi$).

In the following, it is demonstrated how we determined the real confluence angle of a bridging vein in the concrete case in one of the Views 1, 2, or 3:

We determined the sphere point E, which is closest to the observer, by applying the radius r determined from View 1 (image plane parallel to the horizontal) as half the brain's sagittal diameter from the horizon point HP. We were able to do this because we created all views with the same magnification factor. Then we determined the distance $\overline{M'O}$ and the image angle φ' (see Fig. A.10).

We calculated the angle ξ between the original and the image planes:

$$\xi = \sin^{-1}\left(\frac{\overline{M'O}}{r}\right)$$

We then calculated the real confluence angle φ:

$$\varphi = \cos^{-1}\left(\frac{1}{\sqrt{1+\tan^2\varphi'\cdot\left(1-\sin^2\xi\right)}}\right)$$

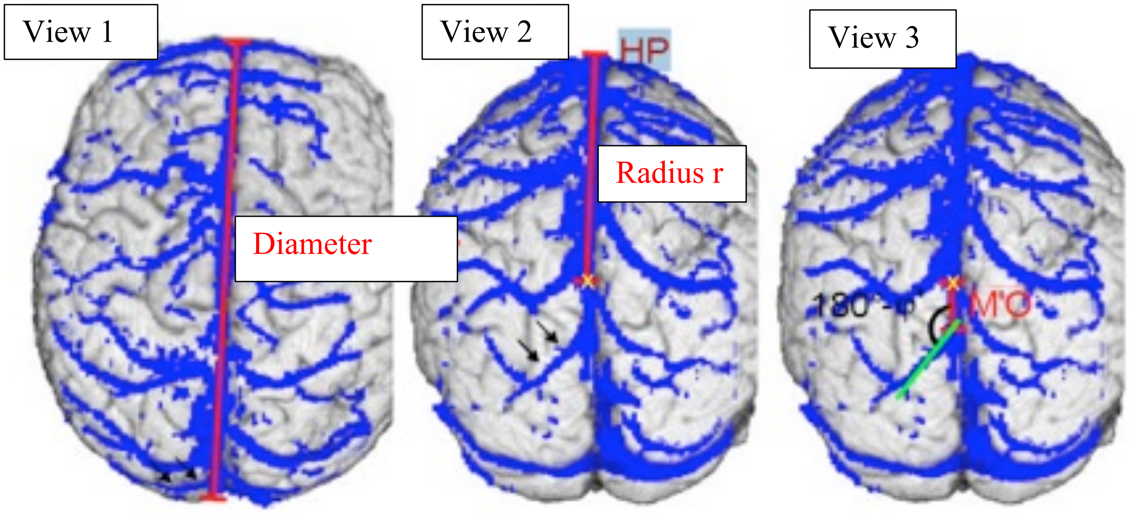

Fig. A.10: Procedure for measuring the confluence angle of an occipital bridging vein (black arrows).

After determining the diameter in View 1, in View 3 the radius r *is calculated as half the diameter, starting from the horizon point HP. From the point* M′ *(yellow cross) thus determined, the distance*

$\overline{M'O}$ *is measured to the intersection between the straight line passing along the middle of the bridging vein (green) and the SSS.*

Together with the measured value φ′*, the values are substituted in the formulas above, giving the real confluence angle* φ.

In this specific case φ′ *was 40.6°, and therefore* φ *was 39.7°.*
